# Association between oropharyngeal dysphagia and functional constipation in older adults: a cross-sectional study

**DOI:** 10.3389/fpubh.2026.1856568

**Published:** 2026-07-03

**Authors:** Yu Wang, Handi Jia, Yiwen Liu, Mengxin Wang, Chenyue Xia, Shuyu Ma, Xiang Zhu, Hongyan Liu, Liugen Wang, Xing’na Zhao, Xi Zeng, Jinhui Gu

**Affiliations:** 1 Department of Rehabilitation Medicine, The People’s Hospital of Suzhou New District, Suzhou, China; 2 Anorectal Department, Suzhou Hospital of Traditional Chinese Medicine Affiliated to Nanjing University of Chinese Medicine, Suzhou, China; 3 Department of Neurology, The People's Hospital of Suzhou New District, Suzhou, China; 4 Respiratory and Critical Care Medicine Department, The Second Medica Center and National Clinical Research Center for Geriatric Diseases, Chinese PLA General Hospital, Beijing, China; 5 Department of Rehabilitation Medicine, First Affiliated Hospital of Zhengzhou University, Zhengzhou, China; 6 Institute of Dysphagia Research, Zhengzhou University, Zhengzhou, China

**Keywords:** ageing, deglutition, deglutition disorders, gastrointestinal disorders, prebyphagia

## Abstract

**Background:**

Oropharyngeal dysphagia and functional constipation are common in older adults, but their relationship remains unclear. This study aims to explore the association between these two issues.

**Methods:**

A cross-sectional study among community-dwelling adults aged ≥65 years was conducted from June to August 2024 in China. Participants were included using cluster random sampling, and data were collected using onsite physical examinations and questionnaires. Oropharyngeal dysphagia and functional constipation were assessed using the Volume-Viscosity Swallow Test and ROME IV Criteria, respectively. Covariates included sociodemographic characteristics, lifestyle factors, and disease information. Univariate analysis and multivariate logistic regression were used to identify confounding variables in the dysphagia-constipation relationship. Propensity Score Matching (PSM) was performed to reduce potential bias and to investigate the net effect.

**Results:**

Totally, 5,581 participants were included. The prevalence of oropharyngeal dysphagia and functional constipation was 28.82% (95% CI: 27.93–30.01%) and 23.95% (95% CI: 22.83–25.07%), respectively. Multivariable regression showed that oropharyngeal dysphagia was significantly associated with functional constipation (aOR = 1.595, 95% CI: 1.390–1.830), and identified 17 PSM covariates. After matching, there were no significant differences in the covariates, and a 7.49% difference was revealed in the functional constipation rate between participants with and without oropharyngeal dysphagia (32.00% vs. 24.08%, *p* < 0.001).

**Conclusion:**

In community-dwelling older adults, oropharyngeal dysphagia and functional constipation were significantly associated and affected approximately one-quarter of individuals, respectively.

## 1 Introduction

Older adults typically experience functional decline, one of which is oropharyngeal dysphagia. (1) It refers to any abnormality in the physiology of swallowing in the upper gastrointestinal tract. (2) Oropharyngeal dysphagia is common in various neurological or respiratory diseases and structural changes. (1) Moreover, ageing can lead to degeneration of oropharyngeal muscles and nerve function. (3) It has been reported that oropharyngeal dysphagia affects approximately one-third of community-dwelling older individuals. (4) Oropharyngeal dysphagia not only limits oral intake and reduces swallowing efficiency but also significantly increases the risk of many adverse outcomes, such as aspiration pneumonia, malnutrition, and electrolyte imbalances. (1, 3, 5, 6) Additionally, studies have shown that dysphagia can affect social functions and cause psychological burden. (7, 8) Therefore, oropharyngeal dysphagia is considered to have multidimensional impacts on quality of life. (9, 10)

Functional constipation is another prevalent health challenge in older adults, (11) affecting approximately one-fifth of the population. (12) It is characterized by difficult stool passage without other secondary causes. (13) Its related symptoms typically include straining during defecation, hard stools, infrequent bowel movements, and bloating. (14) Constipation has been widely recognized to affect physical health and social and psychological well-being. (15) However, since it involves privacy and intimacy, the affected individuals may be reluctant to express it. (14) Consequently, functional constipation is frequently overlooked until it becomes a serious issue. (14)

There may be biological links between oropharyngeal dysphagia and functional constipation. First, dysphagia can restrict oral intake and alter food consistency. Older adults with swallowing difficulty may avoid hard, dry, or fiber-rich foods and may rely more on soft, liquid, or processed diets, which can reduce dietary fiber intake and impair stool formation. (16–18) Second, fear of choking and reduced swallowing safety may lead to insufficient fluid intake, resulting in dehydration, harder stools, and reduced bowel movement frequency. (16, 17) Third, dysphagia is closely related to malnutrition, sarcopenia, and frailty, which may reduce both swallowing muscle reserve and abdominal or pelvic floor muscle function involved in defecation. (19–21) In addition, ageing-related neuromuscular decline, autonomic dysfunction, reduced physical activity, and psychological distress may contribute to both impaired swallowing and gastrointestinal dysmotility. (22–25) Therefore, oropharyngeal dysphagia and functional constipation may not only coexist as two common geriatric problems, but may also be biologically connected through nutritional, muscular, hydration-related, and neurofunctional pathways. However, there is currently insufficient evidence to support this hypothesis, and there is a lack of data on the prevalence of functional constipation in central China. Given the potential impact of these two symptoms on healthy ageing, filling these knowledge gaps is expected to raise awareness and promote early interventions.

Based on this, the main objective of the current study was to explore the association between oropharyngeal dysphagia and functional constipation in older adults. Our secondary objective was to uncover the prevalence of these two symptoms in central China.

## 2 Methods

### 2.1 Participants and procedures

The current research complied with the Declaration of Helsinki and was approved by the Institutional Review Board of the Zhengzhou University First Affiliated Hospital (Approval number: 2024-KY-0323). Written informed consent was obtained from all participants. This cross-sectional study was conducted between June and August 2024 in Zhengzhou, China. Before the investigation began, a sample size estimation was performed using the following formula:


$$n=\frac{z_{a/2}\times p\;(1-p)}{d^{2}}$$

The prevalence of functional constipation was used to estimate the target sample size, which was reported to be 13.6% in older adults. (12) Therefore, in this formula, *p* = 0.136, Type I error probability (a) = 0.05, and margin of error (d) = 0.01. Assuming a 15% sample loss rate, at least 5,311 cases were required.

This study was based on the Senior Health Screening Program in Henan Province, which included 132 primary healthcare service centers to cover older individuals aged 65 years and above across the city, providing them with annual physical examinations. The inclusion criteria were: (1) age 65 years or older, (2) residence in Zhengzhou for more than 2.5 years, and (3) clear consciousness and ability to cooperate with the questionnaire and health examination. Older individuals with severe emotional or psychiatric disorders were excluded.

Cluster random sampling was used. Zhengzhou includes 16 administrative districts, and all districts were included to improve geographic coverage rather than selecting only a subset of districts. In each district, one primary healthcare service center was randomly selected from the local list of centers as the sampling site, resulting in 16 sampling sites. Within each selected center, participants were randomly selected from the registered list of older adults aged ≥65 years. The number of participants selected from each district was proportional to the number of registered older adults, because the same sampling fraction was applied across districts. Specifically, 0.6% of registered older adults in each selected district were randomly sampled. Selected participants were contacted by telephone or home visit. If contact could not be made before the study began, another eligible older adult was randomly selected from the same sampling pool as a replacement. After data collection began, no further sample replacement was performed. Participants were instructed to the corresponding primary healthcare service centers to undergo the routine annual health examinations and complete the questionnaire, along with simple screenings. If participants had mobility issues and were unable to attend, researchers would conduct home visits to collect data with their consent. The specific objective of the study was not disclosed to the participants until they had completed the research to minimize potential response bias. All participants were allowed to voluntarily quit the study. Older adults might have lower educational levels or experience hearing loss, so we allowed researchers to read and explain the questionnaire items. Researchers were trained in advance to avoid any guiding effects. Dual data entry was used to ensure accuracy.

### 2.2 Assessments

#### 2.2.1 Oropharyngeal dysphagia

Considering the potential decrease in patience among older adults and the risk of response bias in self-reports, any self-administered questionnaire was not adopted for dysphagia screening. In the current research, the Volume-Viscosity Swallow Test (V-VST) was used to assess oropharyngeal dysphagia. (26) This assessment has been recommended by relevant guidelines and is widely applied in clinical and community settings due to its good accuracy and specificity. (1, 27, 28) Participants were instructed to swallow 5, 10, and 20 mL of nectar viscosity bolus, liquid, and pudding, respectively. The sequence and logic of swallowing have been described in previous studies. (26) All boluses were prepared using drinkable water (Ice Dew, Coca-Cola, Inc., China) and thickening agents (9000-69-5, Xi’an Hongyao Pharmaceutical Excipients Co., Ltd., China). Based on the V-VST, participants were categorized into non- and oropharyngeal dysphagia groups based on indicators related to safety impairment (coughing, change in voice quality, blood oxygen saturation drop ≥3%) and effectiveness impairment (lip closure, oral residue, multiple swallows, pharyngeal residue). Specifically, blood oxygen saturation was measured using fingertip pulse oximeters (YBK303, Youbaikang Medical Devices Co., Ltd., China). Each participant was assessed by two speech therapists.

#### 2.2.2 Functional constipation

The Rome IV Criteria were used to assess functional constipation. (29) The criteria have been widely applied in the diagnosis of functional gastrointestinal disorders, with good cross-cultural adaptability in multiple regions, including China. (30) The criteria focused on various aspects of bowel movements. Under the circumstance that irritable bowel syndrome has not been diagnosed, participants who met at least two of the criteria were diagnosed with functional constipation. (29)

#### 2.2.3 Covariates

Covariates were selected *a priori* based on previous literature, clinical plausibility, and the potential for confounding the association between oropharyngeal dysphagia and functional constipation. We considered variables that may be associated with dysphagia, constipation, or both conditions, including sociodemographic characteristics, chronic diseases, neurological and cardiopulmonary conditions, nutritional status, physical function, oral/dental status, psychological symptoms, body composition, lifestyle factors, and dietary or fluid-intake behaviors. These covariates were selected before the main statistical analysis rather than solely according to statistical significance. The definitions, assessments, and reasons for including each covariate are shown in Appendix 1. Covariates included: (a) Age, (b) Sex, (c) Permanent residency, (d) Ethnicity, (e) Educational level, (f) Marriage, (g) Hypertension, (h) Diabetes, (i) Physical disability, (j) Dyslipidemia, (k) Neurological disorders, (l) Kidney diseases, (m) Heart diseases, (n) Chronic obstructive pulmonary disease, (31) (o) Nutritional risk, (32) (p) Basic activities of daily living, (33) (q) Missing teeth but no dentures, (r) Body mass index classification, (s) Anxiety symptoms, (34) (t) Exercise frequency, (u) Daily fruit intake, (v) Daily water intake, (w) Sweets or fried foods, (x) High oil or high salt diet, (y) Currently smoking (z) Currently drinking.

### 2.3 Statistical analysis

Categorical data are presented using frequencies and proportions [*n* (%)], and Chi-square tests were used to assess univariate differences. Continuous data were presented using means and standard deviations (x ± s), and *t*-tests were used to assess univariate differences, as no skewed data were included in this study. Participants were first divided into groups based on whether or not they had oropharyngeal dysphagia or functional constipation, respectively. Univariate analysis was conducted within each pair of groups, and statistically significant variables were included in logistic regression to explore the factors associated with the relationship between oropharyngeal dysphagia and functional constipation. For this, each logistic regression model included two key variables. For example, in the model in which oropharyngeal dysphagia was the dependent variable, functional constipation was included as an independent variable, and vice versa.

Propensity Score Matching (PSM) was used to balance potential bias and explore the net effect of oropharyngeal dysphagia and functional constipation. (35) Covariates with significance in any logistic regression were included in the PSM covariate set. Probit regression was used to calculate the propensity scores. Then, cases with and without oropharyngeal dysphagia were handled using one-to-one matching without replacement within a predefined propensity score radius (caliper = 0.1). We used balance tests to assess matching quality. A standardized bias between ±0.1 and no significant univariate differences indicated good matching quality. (36) Univariate differences here were assessed using *t*-tests, as *t*-tests are stricter. Additionally, in kernel density plots, the proximity of the two curves indicated a reduction in potential differences. We also adjusted the matching method using PSM with replacement to test robustness. Data with missing key variables were excluded and with missing partial covariates were handled using multiple imputations. We conducted statistical analyses using STATA 17.0 (Stata Corp., College Station, TX, United States). The significance level in the tests was considered to be 0.05.

## 3 Results

### 3.1 Participant information

Totally, 6,498 older individuals were invited. Of them, 452 did not attend, 218 were unwilling to sign the informed consent after arrival, 59 were excluded due to severe emotional or psychiatric disorders, 165 quitted the study midway, and 23 were excluded due to missing key variables. Ultimately, 5,581 cases were included in the analysis, which was higher than the estimated sample size. Among the participants, 72.82% were aged <75 years, and 55.92% were female. The prevalence of oropharyngeal dysphagia and functional constipation was 28.82% (95% CI: 27.93–30.01%) and 23.95% (95% CI: 22.83–25.07%), respectively. The prevalence of functional constipation was significantly higher in participants with oropharyngeal dysphagia that than in those without [(526/1,609, 32.69%) vs. (811/3,972, 20.42%), *p* < 0.001]. The details of the participants are shown in Table 1.

**Table 1:** Participant characteristics.

Variable		Total (*n* = 5,581)	Functional constipation		*p*	Oropharyngeal dysphagia		*p*
			No (*n* = 4,244)	Yes (*n* = 1,337)		No (*n* = 3,972)	Yes (*n* = 1,609)	
Age [years, *n* (%)]	<75	4,064 (72.82)	3,177 (74.86)	887 (66.34)	<0.001***	2,961 (74.55)	1,103 (68.55)	<0.001***
	≥75	1,517 (27.18)	1,067 (25.14)	450 (33.66)		1,011 (25.45)	506 (31.45)	
Sex [*n* (%)]	Female	3,121 (55.92)	2,341 (55.16)	780 (58.34)	0.041*	2,246 (56.55)	875 (54.38)	0.140
	Male	2,460 (44.08)	1,903 (44.84)	557 (41.66)		1,726 (43.45)	734 (45.62)	
Permanent residency [*n* (%)]	Rural	2,007 (35.96)	1,556 (36.66)	451 (33.73)	0.051	1,427 (35.93)	580 (36.05)	0.932
	Urban	3,574 (64.04)	2,688 (63.34)	886 (66.27)		2,545 (64.07)	1,029 (63.95)	
Ethnicity [*n* (%)]	Others	46 (0.82)	26 (0.61)	20 (1.50)	0.002**	31 (0.78)	15 (0.93)	0.570
	Han	5,535 (99.18)	4,218 (99.39)	1,317 (98.50)		3,941 (99.22)	1,594 (99.07)	
Educational level [*n* (%)]	Primary school or below	2,459 (44.06)	1,855 (43.71)	604 (45.18)	0.346	1,725 (43.43)	734 (45.62)	0.136
	Middle school or above	3,122 (55.94)	2,389 (56.29)	733 (54.82)		2,247 (56.57)	875 (54.38)	
Marriage [*n* (%)]	Others	4,290 (76.87)	3,277 (77.21)	1,013 (75.77)	0.273	3,064 (77.14)	1,226 (76.20)	0.449
	Married	1,291 (23.13)	967 (22.79)	324 (24.23)		908 (22.86)	383 (23.80)	
Hypertension [*n* (%)]	No	2,711 (48.58)	2,114 (49.81)	597 (44.65)	0.001**	1,974 (49.70)	737 (45.80)	0.008**
	Yes	2,870 (51.42)	2,130 (50.19)	740 (55.35)		1,998 (50.30)	872 (54.20)	
Diabetes [*n* (%)]	No	3,614 (64.76)	2,772 (65.32)	842 (62.98)	0.119	2,590 (65.21)	1,024 (63.64)	0.268
	Yes	1,967 (35.24)	1,472 (34.68)	495 (37.02)		1,382 (34.79)	585 (36.36)	
Physical disability [*n* (%)]	No	5,351 (95.88)	4,059 (95.64)	1,292 (96.63)	0.111	3,803 (95.75)	1,548 (96.21)	0.430
	Yes	230 (4.12)	185 (4.36)	45 (3.37)		169 (4.25)	61 (3.79)	
Body mass index classification [kg/m^2^, *n* (%)]	<18.5	130 (2.33)	85 (2.00)	45 (3.37)	<0.001***	58 (1.46)	72 (4.47)	<0.001***
	18.5–24	2,680 (48.02)	2,096 (49.39)	584 (43.68)		1,917 (48.26)	763 (47.42)	
	>24 but <28	2,053 (36.79)	1,570 (36.99)	483 (36.13)		1,487 (37.44)	566 (35.18)	
	≥28	718 (12.87)	493 (11.62)	225 (16.83)		510 (12.84)	208 (12.93)	
Anxiety symptoms [*n* (%)]	No	4,694 (84.11)	3,610 (85.06)	1,084 (81.08)	0.001***	3,382 (85.15)	1,312 (81.54)	0.001**
	Yes	887 (15.89)	634 (14.94)	253 (18.92)		590 (14.85)	297 (18.46)	
Exercise frequency [n (%)]	Almost never	2,639 (47.29)	2,067 (48.70)	572 (42.78)	<0.001***	1,872 (47.13)	767 (47.67)	0.183
	Occasionally	414 (7.42)	255 (6.01)	159 (11.89)		280 (7.05)	134 (8.33)	
	More than once a week	802 (14.37)	675 (15.90)	127 (9.50)		590 (14.85)	212 (13.18)	
	Almost every day	1,726 (30.93)	1,247 (29.38)	479 (35.83)		1,230 (30.97)	496 (30.83)	
Daily fruit intake [*n* (%)]	Sufficient	1,480 (26.52)	1,150 (27.10)	330 (24.68)	0.081	1,066 (26.84)	414 (25.73)	0.396
	Insufficient	4,101 (73.48)	3,094 (72.90)	1,007 (75.32)		2,906 (73.16)	1,195 (74.27)	
Daily water intake [*n* (%)]	Sufficient	1,902 (34.08)	1,581 (37.25)	321 (24.01)	<0.001***	1,433 (36.08)	469 (29.15)	<0.001***
	Insufficient	3,679 (65.92)	2,663 (62.75)	1,016 (75.99)		2,539 (63.92)	1,140 (70.85)	
Sweets or fried foods [*n* (%)]	Less than once a month	3,478 (62.32)	2,786 (65.65)	692 (51.76)	<0.001***	2,513 (63.27)	965 (59.98)	0.025*
	Once a week to once a month	1,517 (27.18)	1,066 (25.12)	451 (33.73)		1,066 (26.84)	451 (28.03)	
	> once a week	586 (10.50)	392 (9.24)	194 (14.51)		393 (9.89)	193 (12.00)	
High oil or high salt diet [*n* (%)]	No	4,246 (76.08)	3,259 (76.79)	987 (73.82)	0.026*	3,077 (77.47)	1,169 (72.65)	<0.001***
	Yes	1,335 (23.92)	985 (23.21)	350 (26.18)		895 (22.53)	440 (27.35)	
Currently smoking [*n* (%)]	No	5,215 (93.44)	3,980 (93.78)	1,235 (92.37)	0.070	3,733 (93.98)	1,482 (92.11)	0.010*
	Yes	366 (6.56)	264 (6.22)	102 (7.63)		239 (6.02)	127 (7.89)	
Currently drinking [*n* (%)]	No	5,223 (93.59)	3,985 (93.90)	1,238 (92.60)	0.090	3,727 (93.83)	1,496 (92.98)	0.238
	Yes	358 (6.41)	259 (6.10)	99 (7.40)		245 (6.17)	113 (7.02)	
Dyslipidemia [*n* (%)]	No	3,767 (67.50)	2,882 (67.91)	885 (66.19)	0.243	2,661 (66.99)	1,106 (68.74)	0.208
	Yes	1,814 (32.50)	1,362 (32.09)	452 (33.81)		1,311 (33.01)	503 (31.26)	
Neurological disorders [*n* (%)]	No	4,676 (83.78)	3,552 (83.69)	1,124 (84.07)	0.746	3,417 (86.03)	1,259 (78.25)	<0.001***
	Yes	905 (16.22)	692 (16.31)	213 (15.93)		555 (13.97)	350 (21.75)	
Kidney diseases [*n* (%)]	No	5,531 (99.10)	4,208 (99.15)	1,323 (98.95)	0.501	3,937 (99.12)	1,594 (99.07)	0.854
	Yes	50 (0.90)	36 (0.85)	14 (1.05)		35 (0.88)	15 (0.93)	
Heart diseases [*n* (%)]	No	5,304 (95.04)	4,054 (95.52)	1,250 (93.49)	0.003**	3,818 (96.12)	1,486 (92.36)	<0.001***
	Yes	277 (4.96)	190 (4.48)	87 (6.51)		154 (3.88)	123 (7.64)	
Chronic obstructive pulmonary disease [*n* (%)]	No	4,793 (85.88)	3,678 (86.66)	1,115 (83.40)	0.003**	3,459 (87.08)	1,334 (82.91)	<0.001***
	Yes	788 (14.12)	566 (13.34)	222 (16.60)		513 (12.92)	275 (17.09)	
Nutritional risk [*n* (%)]	No	3,827 (68.57)	3,083 (72.64)	744 (55.65)	<0.001***	2,867 (72.18)	960 (59.66)	<0.001***
	Yes	1,754 (31.43)	1,161 (27.36)	593 (44.35)		1,105 (27.82)	649 (40.34)	
Basic activities of daily living [*n* (%)]	Impaired	633 (11.34)	483 (11.38)	150 (11.22)	0.871	443 (11.15)	190 (11.81)	0.484
	Normal	4,948 (88.66)	3,761 (88.62)	1,187 (88.78)		3,529 (88.85)	1,419 (88.19)	
Missing teeth but no dentures [*n* (%)]	No	4,377 (78.43)	3,353 (79.01)	1,024 (76.59)	0.061	3,158 (79.51)	1,219 (75.76)	0.002**
	Yes	1,095 (21.49)	934 (21.20)	161 (23.33)		812 (20.26)	283 (26.01)	
Waist size (cm, x ± *t*)		84.88 ± 9.63	84.47 ± 9.43	86.19 ± 10.14	<0.001***	84.95 ± 9.63	84.71 ± 9.63	0.397

^*^p < 0.05, ^**^p < 0.01, ^***^p < 0.001.

### 3.2 Investigation of potential confounding covariates

In each pair of the subgroups (non- vs. constipation, non- vs. dysphagia), variables that showed statistical significance in the univariate analysis were included in the logistic regression, as shown in Table 2. After adjusting for confounders, oropharyngeal dysphagia was found to be significantly associated with functional constipation (aOR = 1.595, 95% CI: 1.390–1.830). Additionally, the following variables were considered as confounding covariates (*p* < 0.05) in the relationship between oropharyngeal dysphagia and functional constipation and were included in the PSM covariate set: age, sex, ethnicity, waist size, body mass index classification <18.5, body mass index classification ≥28, anxiety symptoms, exercise frequency, daily water intake, sweets or fried foods, high oil or high salt diet, currently smoking, neurological disorders, heart diseases, chronic obstructive pulmonary disease, nutritional risk, and missing teeth but no dentures.

**Table 2:** Multiple logistic regressions to identify potential confounding covariates.

Variable		Model I: independent variable: functional constipation			Model II: independent variable: oropharyngeal dysphagia		
		aOR	95% CI	*p*	aOR	95% CI	*p*
Another key variable	No	(Ref.)			(Ref.)		
	Yes	1.595	1.390–1.830	<0.001***	1.593	1.388–1.827	<0.001***
Age	<75	(Ref.)			(Ref.)		
	≥75	1.466	1.275–1.686	<0.001***	1.238	1.085–1.413	0.002**
Sex	Female	(Ref.)			NA		
	Male	0.821	0.719–0.938	0.004*			
Ethnicity	Others	(Ref.)			NA		
	Han	0.404	0.216–0.754	0.004*			
Hypertension	No	(Ref.)			(Ref.)		
	Yes	1.092	0.953–1.252	0.204	1.090	0.960–1.237	0.183
Waist size		1.020	1.012–1.029	<0.001***	NA		
Body mass index classification	<18.5	1.886	1.262–2.817	0.002**	2.884	1.994–4.172	<0.001***
	18.5–24	(Ref.)			(Ref.)		
	>24 but <28	0.981	0.840–1.145	0.805	0.936	0.820–1.069	0.327
	≥28	1.273	1.009–1.604	0.041*	0.961	0.795–1.162	0.684
Anxiety symptoms	No	(Ref.)			(Ref.)		
	Yes	1.278	1.079–1.513	0.004**	1.266	1.081–1.485	0.003**
Exercise frequency		1.115	1.060–1.171	<0.001***	NA		
Daily water intake	Sufficient	(Ref.)			(Ref.)		
	Insufficient	1.772	1.533–2.048	<0.001***	1.268	1.113–1.443	<0.001***
Sweets or fried foods		1.411	1.289–1.544	<0.001***	1.039	0.951–1.135	0.394
High oil or high salt diet	No	(Ref.)			(Ref.)		
	Yes	1.202	1.031–1.402	0.019*	1.192	1.039–1.368	0.012*
Currently smoking	No	NA			(Ref.)		
	Yes				1.358	1.076–1.713	0.010*
Neurological disorders	No	NA			(Ref.)		
	Yes				1.676	1.434–1.957	<0.001***
Heart diseases	No	(Ref.)			(Ref.)		
	Yes	1.261	0.957–1.663	0.100	1.723	1.334–2.224	<0.001***
Chronic obstructive pulmonary disease	No	(Ref.)			(Ref.)		
	Yes	1.099	0.911–1.325	0.323	1.340	1.127–1.594	0.001**
Nutritional risk	No	(Ref.)			(Ref.)		
	Yes	1.938	1.697–2.214	<0.001***	1.593	1.405–1.808	<0.001***
Missing teeth but no dentures	No	NA			Ref.)		
	Yes				1.214	1.053–1.400	0.007**

^*^p < 0.05, ^**^p < 0.01, ^***^p < 0.001. aOR adjusted Odds Ratio. Model I was adjusted for oropharyngeal dysphagia, age, sex, ethnicity, hypertension, waist size, body mass index classification, anxiety symptoms, exercise frequency, daily water intake, sweets or fried foods, high oil or high salt diet, heart diseases, chronic obstructive pulmonary disease, nutrition risk. Model II was adjusted for functional constipation, age, hypertension, body mass index classification, anxiety symptoms, daily water intake, sweets or fried foods, high oil or high salt diet, currently smoking, neurological disorders, heart diseases, chronic obstructive pulmonary disease, nutrition risk, and missing teeth but no dentures.

### 3.3 Matching results and quality

**Figure 1:**
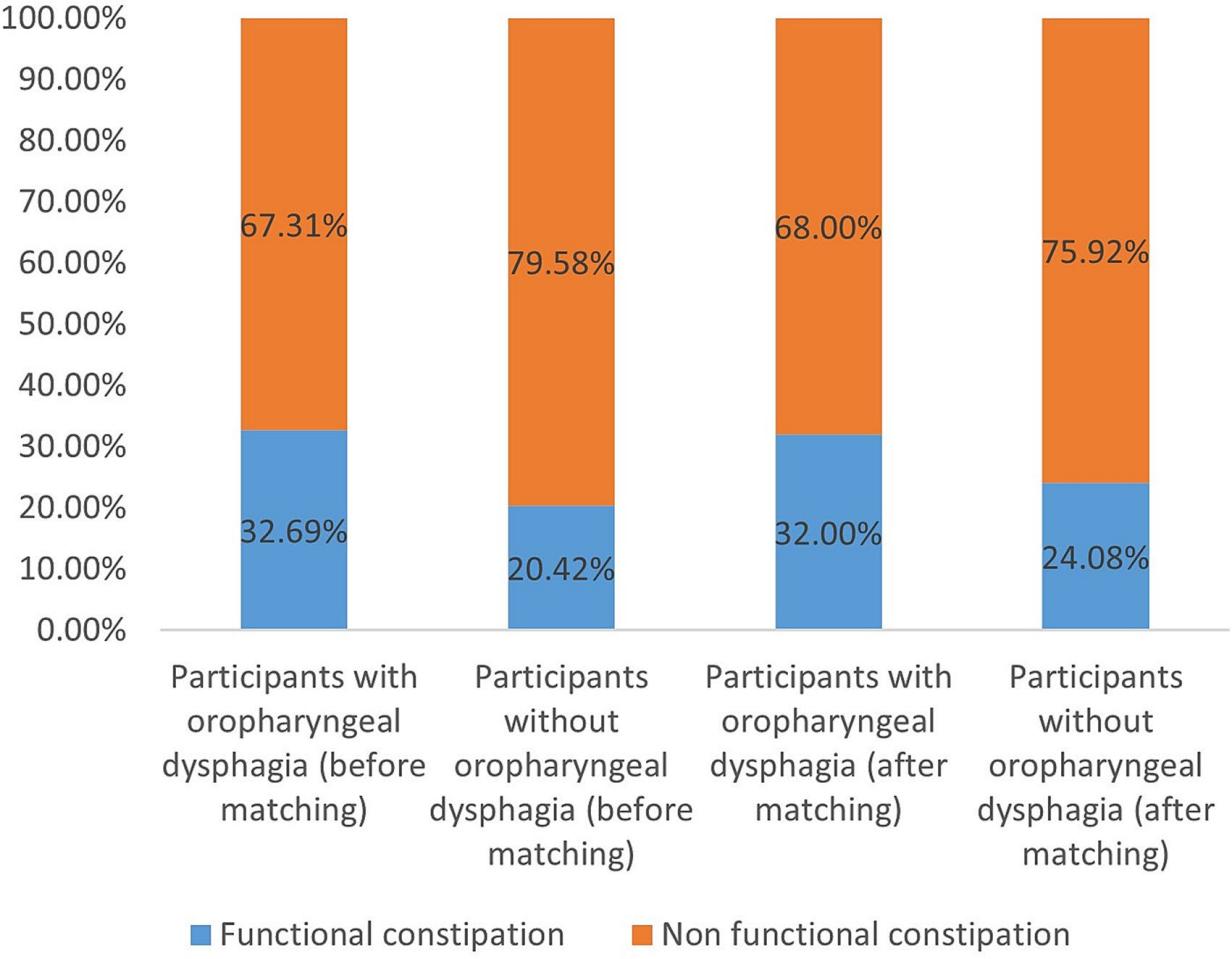
The prevalence of functional constipation between participants with and without oropharyngeal dysphagia before and after matching.

The PSM captured 1,578 pairs of samples with and without oropharyngeal dysphagia. After matching, no statistical significance was found in any of the PSM covariates in the univariate analysis, and all standardized biases were within ±10%, as shown in Table 3. The kernel density plots are shown in Appendix 2. These indicated a good matching quality. After PSM, the participants with oropharyngeal dysphagia showed a 7.92% higher prevalence of functional constipation than their counterparts without oropharyngeal dysphagia (32.00% vs. 24.08%, *p* < 0.001), as shown in Figure 1. We adjusted the matching method to with-replacement PSM and obtained similar results, which supported the robustness of our findings. The balance test for the second matching is shown in Appendix 3.

**Table 3:** Propensity score matching balance test.

Variable		Treated	Control	Standardized bias	Degree of standardized bias reduction	*t*	*p*
Age	Before matching	0.314	0.255	13.31%	91.81%	4.446	<0.001***
	After matching	0.311	0.316	−1.09%		−0.307	0.759
Sex	Before matching	0.456	0.435	4.35%	91.24%	1.472	0.141
	After matching	0.455	0.457	−0.38%		−0.107	0.915
Ethnicity	Before matching	0.991	0.992	−1.65%	61.00%	−0.547	0.584
	After matching	0.990	0.990	0.64%		0.180	0.857
Waist size	Before matching	84.708	84.949	−2.50%	36.20%	−0.846	0.397
	After matching	84.818	84.971	−1.60%		−0.448	0.654
Body mass index classification <18.5	Before matching	0.045	0.015	17.83%	93.94%	5.485	<0.001***
	After matching	0.033	0.031	1.08%		0.303	0.762
Body mass index classification ≥28	Before matching	0.129	0.128	0.26%	27.83%	0.088	0.930
	After matching	0.131	0.130	0.19%		0.053	0.958
Anxiety symptoms	Before matching	0.185	0.149	9.68%	66.52%	3.218	0.001**
	After matching	0.182	0.195	−3.24%		−0.911	0.363
Exercise frequency	Before matching	2.272	2.297	−1.88%	−59.44%	−0.635	0.525
	After matching	2.258	2.298	−2.99%		−0.841	0.400
Daily water intake	Before matching	0.709	0.639	14.82%	98.12%	5.074	<0.001***
	After matching	0.707	0.706	0.28%		0.078	0.938
Sweets or fried foods	Before matching	0.520	0.466	7.88%	94.22%	2.641	0.008**
	After matching	0.511	0.508	0.46%		0.128	0.898
High oil or high salt diet	Before matching	0.273	0.225	11.14%	88.53%	3.719	<0.001***
	After matching	0.271	0.277	−1.28%		−0.359	0.720
Currently smoking	Before matching	0.079	0.060	7.38%	93.59%	2.433	0.015*
	After matching	0.077	0.079	−0.47%		−0.133	0.894
Neurological disorders	Before matching	0.218	0.140	20.41%	97.71%	6.668	<0.001***
	After matching	0.209	0.211	−0.47%		−0.131	0.896
Heart diseases	Before matching	0.076	0.039	16.22%	98.49%	5.161	<0.001***
	After matching	0.072	0.072	0.25%		0.069	0.945
Chronic obstructive pulmonary disease	Before matching	0.171	0.129	11.71%	73.63%	3.870	<0.001***
	After matching	0.169	0.157	3.09%		0.868	0.386
Nutritional risk	Before matching	0.403	0.278	26.63%	98.54%	8.845	<0.001***
	After matching	0.393	0.391	0.39%		0.109	0.913
Missing teeth but no dentures	Before matching	0.242	0.205	8.99%	95.07%	3.006	0.003**
	After matching	0.241	0.243	−0.44%		−0.125	0.901

^*^p < 0.05, ^**^p < 0.01, ^***^p < 0.001.

## 4 Discussion

Based on this on-site investigation, oropharyngeal dysphagia appears to be significantly associated with functional constipation in older adults. These functional disorders are prevalent and severely impact the quality of life. Uncovering this relationship is expected to provide references for prevention and intervention, which is of practical significance. The current study found that the prevalence of oropharyngeal dysphagia was 28.82% among participants. A study based on provincial data in China reported a prevalence of 22.06%, (7) and another Japanese study reported 20.4%, (37) both lower than our results. A potential reason might be that these studies used the 10-item Eating Assessment Tool to assess swallowing function (38). A systematic review showed that this self-reported questionnaire could produce lower positive rates (4). Therefore, this study adopted the V-VST, which involves both swallowing efficiency and safety, and could reduce response bias (26). A meta-analysis found that oropharyngeal dysphagia affected approximately 30.52% of older individuals, similar to our findings (4). The prevalence of functional constipation was relatively low (23.95%). A review showed that constipation affected 16–32% of older adults (11), consistent with our results. However, another global analysis reported a lower prevalence (12). A possible reason might be that our study was conducted in a relatively developed area, where the availability of processed foods and public transportation might have influenced dietary and exercise habits.

There might be a bidirectional causality between oropharyngeal dysphagia and functional constipation. First, dietary fiber is primarily found in foods, such as whole grains, vegetables, fruits, and legumes, which are typically harder or require more time to chew. Individuals with oropharyngeal dysphagia are recommended to choose foods with more acceptable texture and consistency, or to prepare food in liquid form to reduce oral intake risks (39). Therefore, this may decrease fiber intake (18), which in turn affects bowel movements (17). Second, due to the fear of choking, older adults with oropharyngeal dysphagia may reduce their fluid intake, leading to dehydration, dry stools, and worsening constipation (16, 17). Oropharyngeal dysphagia significantly increases the risk of malnutrition and sarcopenia (21), which might affect digestive function and defecation (19). Psychologically, swallowing disorders are associated with anxiety symptoms and social isolation (7, 8). Chronic negative emotions are considered to affect gut function, leading to constipation (23). Conversely, constipation can result in poor appetite and malabsorption, further exacerbating the overall frailty in older adults (11). Frailty is one of the risk factors for oropharyngeal dysphagia, and it may also affect physical activities, further causing functional decline (20). Therefore, oropharyngeal dysphagia and functional constipation might form a vicious cycle. It should be noted that oral frailty or oral hypofunction may be another important explanation for the observed association (40). In older adults, impaired dentition and inadequate prosthetic support are only part of oral functional decline (41). Reduced masticatory performance, weak tongue pressure, impaired tongue–lip motor coordination, oral dryness, and insufficient saliva may also interfere with bolus formation, oral transit, and swallowing efficiency. These oral-phase problems may lead older adults to avoid hard or fiber-rich foods, reduce fluid intake because of fear of choking, or rely on softer and more processed diets, thereby potentially increasing the risk of constipation. In addition, oral frailty is closely related to malnutrition, sarcopenia, and general frailty, which may further link swallowing impairment with reduced gastrointestinal motility and defecation difficulty (20). Therefore, the association observed in this study may partly reflect a broader oral–nutritional–frailty pathway rather than the effect of pharyngeal swallowing impairment alone.

Oropharyngeal dysphagia and functional constipation might share several common risk factors, such as age, medical conditions, emotional issues, and nutritional status, some of which have been validated by our logistic regression. In addition, missing teeth without dentures should be interpreted as a simple dental/prosthetic indicator rather than a comprehensive measure of oral frailty. Oral frailty and oral hypofunction are multidimensional conditions involving not only dentition and prosthetic status, but also tongue strength, masticatory performance, salivary condition, oral motor coordination, and oral hygiene. Therefore, although this variable was included as a covariate in the present analysis, it could not fully capture oral-phase dysfunction or the broader oral functional decline that may affect bolus formation and swallowing efficiency. As people age, their skeletal muscle mass and strength decrease. This not only affects the swallowing-related muscles, decreasing swallowing efficiency, (24) but may also impact the function of the abdominal and pelvic floor muscles, thus affecting the ability to defecate (11, 25). Moreover, ageing and emotional issues have been associated with the autonomic nervous system function, which affects both the coordination of swallowing muscles and gastrointestinal motility and secretion, leading to constipation (22, 42). Interestingly, many studies have suggested that presbyphagia and functional constipation should be considered as geriatric syndromes (11, 25, 43, 44). Nutritional status and physical activities are considered to influence muscle levels and intestinal motility (12, 45, 46). Additionally, many dysphagia-related diseases can increase the risk of constipation (12, 47). The potential reason might be that these diseases affect physical activities, sympathetic excitation, or common pathophysiological mechanisms. Furthermore, some medications used to treat neurological diseases or swallowing-related symptoms might have side effects that lead to constipation (11, 48). After using PSM to adjust for covariates, the prevalence of functional constipation in cases with oropharyngeal dysphagia was significantly higher than that in those without. This indicated that there might be common risk factors or mediating variables affecting both symptoms, which are beyond our current understanding and require further investigation.

This study had some limitations. First, the cross-sectional design hindered causal inferences, and our findings could only remain at the correlation level. Longitudinal designs should be considered in the future to address this issue. Second, this study was conducted in a city in central China, which limited the representativeness of the sample. A larger-scale study would help to enhance the generalizability of our conclusions. Another limitation is that comprehensive oral frailty or oral hypofunction assessments were not performed. Residual confounding related to oral frailty may remain, and the specific contribution of oral-phase dysfunction to the association between oropharyngeal dysphagia and functional constipation could not be fully determined. In addition, we did not use video fluoroscopic swallowing studies, the gold standard for diagnosing oropharyngeal dysphagia. Lastly, we only considered older individuals registered under the Senior Health Screening Program in Henan Province. Although this program has covered over 95% of the local older population, the lack of random sampling from the entire population might have introduced sampling bias.

## Data Availability

The raw data supporting the conclusions of this article will be made available by the authors, without undue reservation.
