# Supplementary material for: Association between oropharyngeal dysphagia and functional constipation in older adults: a cross-sectional study
Source: Front Public Health. 2026 Jul 3;14:1856568. doi: 10.3389/fpubh.2026.1856568 (PMC13376252; doi:10.3389/fpubh.2026.1856568)
Supplement: Supplementary file 1 [file Table_1.DOCX]

Association between Oropharyngeal Dysphagia and Functional Constipation in Older Adults: A Cross-sectional Study

**Supplementary materials**

Appendix 1. The definition, assessments, and reasons of covariates

| Item | Assessment | Definition | Reason |
| --- | --- | --- | --- |
| Age | ID card | Based on ID card birthdate | Age may affect intestinal motility and oropharyngeal nerve and muscle function. |
| Sex | On site | Based on biological sex, rather than self-identified gender | The prevalence of constipation in females has been reported to be higher than in males. |
| Permanent residency | On site | Question: Do you currently live primarily in an urban or rural area? | China's urban-rural dual structure is very distinct, with significant differences in lifestyle, healthcare facilities, and other factors, which may influence health. |
| Ethnicity | ID card | Based on ethnicity shown on the ID card | This is a common demographic variable. Additionally, different ethnic groups may have genetic or lifestyle differences. |
| Educational level | On site | Question: What is your highest educational level? | Educational level may affect health education, self-health management, etc., which could influence health outcomes. |
| Body mass index classification | On site | Calculated based on measurements from a weight scale and height device | Body mass index reflects an individual's body weight, and those who are underweight may have insufficient muscle mass, affecting swallowing and bowel movements. Overweight individuals may also face similar issues. |
| Marriage | On site | Question: What is your current marital status? | This is a common demographic variable. Furthermore, most studies show that married individuals generally have better health compared to others. |
| Hypertension | First based on medical records, a diagnosis within the past five years is considered valid. If the result is negative, the primary care physician responsible will be consulted. | | The three major chronic diseases are considered to be related to many functional impairments and diseases, and are therefore commonly included as background variables. |
| Diabetes |  |  |  |
| Dyslipidemia |  |  |  |
| Physical disability | Home visit or on site | Motor impairments in the limbs significantly affect mobility or result in the absence of any part of limbs. | Individuals with physical disabilities may face issues such as insufficient physical activity and social isolation, which can lower their quality of life and affect both their physical and mental health. |
| Neurological disorders | First based on medical records, If negative, the primary care physician responsible will be consulted. | e.g. stroke and Parkinson's Disease, | Neurological disorders are the main causes of swallowing disorders and may also affect bowel movements through the gut-brain axis. |
| Kidney diseases |  | e.g. kidney failure, acute nephritis, and chronic nephritis | Heart diseases and kidney diseases may indirectly impact swallowing function and intestinal motility by affecting systemic circulation, neurological function, or metabolic processes, thus being associated with swallowing disorders and constipation. |
| Heart diseases |  | e.g. myocardial infarction, angina pectoris, coronary artery revascularization, congestive heart failure and precordial pain |  |
| Chronic obstructive pulmonary disease (COPD) | On site or medical records | The doctor assesses the potential presence of COPD. If suspected, the COPD Screening Questionnaire is administered first. If the result is positive, the individual is defined as having COPD if their FEV1/FVC ratio in post-bronchodilator pulmonary function testing (PFT) is below 0.7. | COPD can significantly affect respiratory rhythm, thereby reducing swallowing safety. It can also impact oxygen intake, which may, in turn, affect bowel movements. |
| Nutrition risk | On site | Mini Nutritional Assessment | Nutritional status is significantly related to both swallowing function and gut function. |
| Basic activities of daily living | On site | Geriatric Self-Care Scale | Swallowing and bowel movements are both associated with the ability to perform activities of daily living. Additionally, elderly individuals with poor self-care abilities are often more frail. |
| Missing teeth but no dentures | On site | Only for the 28 regular teeth, missing teeth without dentures for over a month. | Oral frailty may affect swallowing function, which in turn impacts nutrition and fiber intake |
| Anxiety symptoms | On site | 7-item Genderized Anxiety Disorders Scale | Anxiety can affect pharyngeal muscle function and intestinal motility. |
| Exercise frequency | On site | Question: What is the frequency of your physical exercise? Only activities with intensity greater than brisk walking.   1. Every day 2. More than once a week 3. Occasionally 4. No exercise | Exercise habits influence muscle mass and intestinal motility. Regular physical activity helps maintain muscle strength and mass, which can improve digestive function and promote bowel movements. Conversely, a lack of exercise may lead to muscle atrophy and slower gastrointestinal motility. |
| Daily fruit intake | On site | Question: Is your daily fruit intake less than the weight of one apple? | Fruits contain many fibers, which may influence intestinal motility. |
| Daily water intake | On site | Question: Do you drink more than 8 cups of water a day? | Water intake not only affects bowel movements but may also influence saliva secretion, which in turn affects swallowing function. |
| Sweets or fried foods | On site | Question: How often do you eat processed sweets and fried foods? | Processed and fried foods are believed to affect bowel movements because a large amount of fiber is lost during the processing. |
| High oil or high salt diet | On site | Question: Do you think your usual diet is high in oil or salt? | An unbalanced diet is believed to affect gut microbiota and digestive function. |
| Currently smoking | On site | Question: Do you have the habit of smoking or drinking alcohol in the past six months? | Smoking and drinking are common lifestyle variables. Additionally, smoking may affect oral and throat health, while drinking may influence digestive system function and intestinal motility. |
| Currently drinking |  |  |  |

Appendix 2. Kernel density plots

| 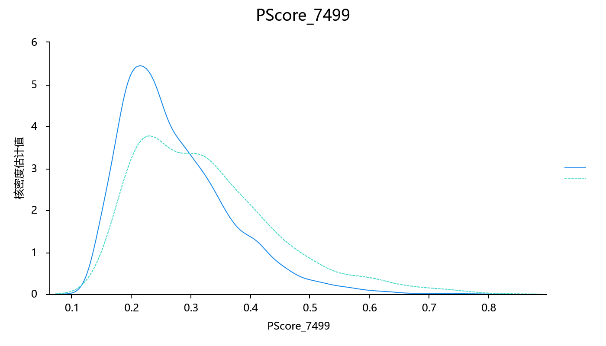 | 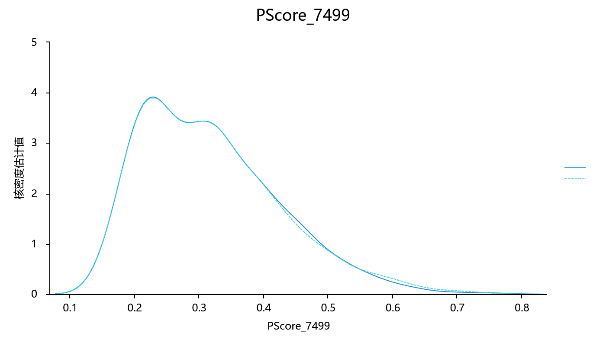 |
| --- | --- |
| Before matching | After matching |

Appendix 3. Balance test for the second matching

| Variable | | Treated | Control | Standardized bias | Degree of standardized bias reduction | t | P |
| --- | --- | --- | --- | --- | --- | --- | --- |
| Age | Before matching | 0.314 | 0.255 | 13.31% | 74.32% | 4.446 | <0.001 |
|  | After matching | 0.314 | 0.299 | 3.42% |  | 0.872 | 0.383 |
| Sex | Before matching | 0.456 | 0.435 | 4.35% | -67.35% | 1.472 | 0.141 |
|  | After matching | 0.456 | 0.420 | 7.29% |  | 1.858 | 0.063 |
| Ethnicity | Before matching | 0.991 | 0.992 | -1.65% | 51.26% | -0.547 | 0.584 |
|  | After matching | 0.991 | 0.990 | 0.80% |  | 0.204 | 0.839 |
| Waist size | Before matching | 84.708 | 84.949 | -2.50% | 7.35% | -0.846 | 0.397 |
|  | After matching | 84.708 | 84.937 | -2.32% |  | -0.587 | 0.557 |
| Body mass index classification <18.5 | Before matching | 0.045 | 0.015 | 17.83% | 63.00% | 5.485 | <0.001 |
|  | After matching | 0.045 | 0.032 | 6.60% |  | 1.683 | 0.093 |
| Body mass index classification ≥28 | Before matching | 0.129 | 0.128 | 0.26% | -1776.37% | 0.088 | 0.930 |
|  | After matching | 0.129 | 0.146 | -4.89% |  | -1.241 | 0.215 |
| Anxiety symptoms | Before matching | 0.185 | 0.149 | 9.68% | 90.60% | 3.218 | 0.001 |
|  | After matching | 0.185 | 0.181 | 0.91% |  | 0.232 | 0.816 |
| Exercise frequency | Before matching | 2.272 | 2.297 | -1.88% | 57.72% | -0.635 | 0.525 |
|  | After matching | 2.272 | 2.261 | 0.79% |  | 0.202 | 0.840 |
| Daily water intake | Before matching | 0.709 | 0.639 | 14.82% | 70.45% | 5.074 | <0.001 |
|  | After matching | 0.709 | 0.688 | 4.38% |  | 1.113 | 0.266 |
| Sweets or fried foods | Before matching | 0.520 | 0.466 | 7.88% | 66.42% | 2.641 | 0.008 |
|  | After matching | 0.520 | 0.502 | 2.65% |  | 0.675 | 0.499 |
| High oil or high salt diet | Before matching | 0.273 | 0.225 | 11.14% | 69.14% | 3.719 | <0.001 |
|  | After matching | 0.273 | 0.258 | 3.44% |  | 0.877 | 0.380 |
| Currently smoking | Before matching | 0.079 | 0.060 | 7.38% | 67.64% | 2.433 | 0.015 |
|  | After matching | 0.079 | 0.073 | 2.39% |  | 0.611 | 0.542 |
| Neurological disorders | Before matching | 0.218 | 0.140 | 20.41% | 78.20% | 6.668 | <0.001 |
|  | After matching | 0.218 | 0.199 | 4.45% |  | 1.137 | 0.256 |
| Heart diseases | Before matching | 0.076 | 0.039 | 16.22% | 84.39% | 5.161 | <0.001 |
|  | After matching | 0.076 | 0.070 | 2.53% |  | 0.647 | 0.517 |
| Chronic obstructive pulmonary disease | Before matching | 0.171 | 0.129 | 11.71% | 85.40% | 3.870 | <0.001 |
|  | After matching | 0.171 | 0.165 | 1.71% |  | 0.436 | 0.663 |
| Nutrition risk | Before matching | 0.403 | 0.278 | 26.63% | 73.73% | 8.845 | <0.001 |
|  | After matching | 0.403 | 0.369 | 7.00% |  | 1.756 | 0.079 |
| Missing teeth but no dentures | Before matching | 0.242 | 0.205 | 8.99% | 49.92% | 3.006 | 0.003 |
|  | After matching | 0.242 | 0.223 | 4.50% |  | 1.151 | 0.250 |

Settings: Matching with replacement; Caliper value = 0.1; number of successful matches = 1609
